# Editorial: Dental caries and periodontal diseases as non-communicable chronic diseases

**DOI:** 10.3389/froh.2022.1113029

**Published:** 2023-01-06

**Authors:** Cassiano Kuchenbecker Rösing, Cameron Randall, Rodrigo A. Giacaman

**Affiliations:** ^1^ School of Dentistry, Federal University of Rio Grande do Sul, Porto Alegre, Brazil; ^2^ University of Washington School of Dentistry, University of Washington, Seattle, WA, United States; ^3^ Faculty of Dentistry, University of Talca, Talca, Chile

**Keywords:** dental caries, periodontal disease, chronic disease, risk factor, oral health promotion

**Editorial on the Research Topic** Dental caries and periodontal diseases as non-communicable chronic diseases

Dental caries and periodontal diseases are the most prevalent oral diseases worldwide. They are considered public health problems, might lead to tooth loss and impair quality of life (1). They share risk factors with chronic diseases such as cancer, cardiovascular problems, diabetes, among others. Smoking, unhealthy diets, overweight, lack of physical exercise increase the risk of all chronic diseases including oral diseases (1).

The Global Burden of Disease study (1) demonstrated that oral conditions affected, in 2010, 3.9 billion people and untreated dental caries was the most prevalent condition (2).

Taking into consideration the presented scenario, two important articles were published in The Lancet (1, 3). The first article emphasizes that oral diseases present serious health and economic consequences and are associated with reduced quality of life (1). After looking to all risk factors with them associated, the article suggests that there is urgent need to consider oral diseases as non-communicable chronic diseases with high priority in terms of global health.

The second article is a call for radical action in order to end the neglect of oral health (3). This study suggests a model from which the presence of disease affects and is affected by health and economical situations and points to the necessity of public policies towards oral health in all aspects including commercial and economic interests. A special emphasis is given to the hazardous effects of high sugar consumption, which is also a risk factor for different other non-communicable chronic diseases.

The present Research Topic comprises three articles, all focusing on the understanding of dental caries and periodontal diseases as chronic diseases. The study by Wolf et al. makes an overview of the relevance of dental caries and periodontal diseases, especially sharing risk factors with other non-communicable chronic diseases. In such reflection, the authors point to the importance of these diseases in economic, social and moral determinants of overall health. In order to better handle such problems, oral health professionals should be trained by interdisciplinary approaches. In such approaches, a paradigm shift is warranted so that health lifestyle is one of the important focuses for prevention and treatment of these diseases. Therefore public health and individual policies need to focus in disseminating the existing knowledge that oral health is part of overall health.

The study by Giacaman et al. that is part of this collection specifically focuses on Dental Caries as a non-communicable disease. The understanding of dental caries as a non-communicable disease is a profound paradigm shift since previous concepts considered dental caries as an infectious (and transmissible) disease. The current understanding supports that the occurrence of dental caries is related to an ecological shift in which oral biofilms become dysbiotic especially due to environmental changes, especially the frequent intake of sugars. The burden of dental caries needs to be controlled by change in modifiable risk factors and in an interdisciplinary approach. Also, it is highlighted that spreading these concepts is of utmost importance among the community (clinicians, stakeholders, researchers and patients).

The the study by Zhang et al. included in the collection is a systematic review with meta-analysis focusing in an important repercussion of periodontal diseases: adverse pregnancy outcomes. It is noteworthy that preterm and/or low birth weight are impacting adverse pregnancy outcomes in health of the infants and are of high costs for health systems. The systematic review is based on case-control and prospective cohort studies, that, when merged, demonstrate that pregnant women with periodontal diseases present higher and significant odds of having preterm birth and low birth weight infants. The results encountered herein also support the need of preventing and treating periodontal diseases in order to achieve better general health.

In summary, this Research Topic emphasizes that understanding dental caries and periodontal diseases as non-communicable chronic diseases changes the way Dentistry approaches these diseases. Approaches that go beyond the mouth are needed, with the help of different professionals (interdisciplinary) and focusing in common-risk factors are needed and have the potential of decreasing the high burden of oral diseases in mankind. This message needs to be communicated for policy makers, individuals responsible for health education (including universities), professional associations so that the community may be positively affected. Strategies focusing on healthy lifestyle need to be emphasized both for individuals and populations.
